# Visual field asymmetries in numerosity processing

**DOI:** 10.3758/s13414-022-02585-1

**Published:** 2022-10-18

**Authors:** Ramakrishna Chakravarthi, Danai Papadaki, Jan Krajnik

**Affiliations:** School of Psychology, Kings College, University of Aberdeen, William Guild Building S7, Aberdeen, AB24 3FX UK

**Keywords:** Subitizing, Estimation, Enumeration, Crowding, Visual field asymmetry, Attention

## Abstract

A small number of objects can be rapidly and accurately enumerated, whereas a larger number of objects can only be approximately enumerated. These *subitizing* and *estimation* abilities, respectively, are both spatial processes relying on extracting information across spatial locations. Nevertheless, whether and how these processes vary across visual field locations remains unknown. Here, we examined if enumeration displays asymmetries around the visual field. Experiment 1 tested small number (1–6) enumeration at cardinal and non-cardinal peripheral locations while manipulating the spacing among the objects. Experiment 2 examined enumeration at cardinal locations in more detail while minimising crowding. Both experiments demonstrated a Horizontal-Vertical Asymmetry (HVA) where performance was better along the horizontal axis relative to the vertical. Experiment 1 found that this effect was modulated by spacing with stronger asymmetry at closer spacing. Experiment 2 revealed further asymmetries: a Vertical Meridian Asymmetry (VMA) with better enumeration on the lower vertical meridian than on the upper and a Horizontal Meridian Asymmetry (HMA) with better enumeration along the left horizontal meridian than along the right. All three asymmetries were evident for both subitizing and estimation. HVA and VMA have been observed in a range of visual tasks, indicating that they might be inherited from early visual constraints. However, HMA is observed primarily in mid-level tasks, often involving attention. These results suggest that while enumeration processes can be argued to inherit low-level visual constraints, the findings are, parsimoniously, consistent with visual attention playing a role in both subitizing and estimation.

**Supplementary Information:**

The online version contains supplementary material available at 10.3758/s13414-022-02585-1.

## Introduction

Humans can quickly enumerate the number of objects in a visual scene. Researchers have identified distinct subsystems of this enumeration process. First, *subitizing* is the ability to rapidly and accurately enumerate a small number of objects (~3–4; Jevons, 1897; Kaufman et al., 1949; Warren, 1897). If there are more objects than this subitizing limit, such objects are either rapidly and approximately *estimated* or laboriously and accurately *counted*. Estimation is thought to be undertaken by the Approximate Number System (Dehaene, 1992), which quickly extracts numerosity using visual features (Burr & Ross, 2008; Dakin et al., 2011; Gebuis et al., 2016). On the other hand, counting is accurate and relies on a host of cognitive facilities such as visual attention, working memory, eye movements, symbolic representations, and linguistic and arithmetic concepts to serially enumerate each object (Cavanagh & He, 2011; Piazza & Eger, 2016). There is considerable debate about whether subitizing and estimation are truly distinct processes (e.g., Anobile et al., 2016; Dehaene, 1992; Feigenson et al., 2004) or a single mechanism whose precision reduces with increasing numerosity (Cai et al., 2021; Cheyette & Piantadosi, 2020; Tsouli et al., 2022).

Irrespective of the underlying mechanisms, it appears that enumeration is a spatial process. In the case of subitizing, previous research has shown that it relies on individuating each object from its background (Mazza et al., 2013; Mazza & Caramazza, 2015; Xu & Chun, 2009). This individuation mechanism is a purely spatial process where the locations of a small number of objects are tagged (Mazza & Caramazza, 2015; Trick & Pylyshyn, 1994). For example, objects of various sizes can be easily individuated if they are presented at different locations, but not if they are concentric (Saltzman & Garner, 1948; Trick & Pylyshyn, 1994). Similarly, when observers are presented with a large number of coloured discs and asked to report the number of colours, performance is highly inefficient when the coloured discs are intermixed, but efficient if they are clustered by colour (Watson et al., 2005). This suggests that location-based segmentation is necessary for subitizing.

Similarly, estimation is a spatial process. First, it has been shown that estimation of objects, at very high numerosities, relies on determining their spatial density, which can be derived from the spatial frequency content of the scene (Dakin et al., 2011). Second, spatial organisation and spatial grouping have been long known to strongly modulate estimation (Franconeri et al., 2009; Ginsburg, 1991; Yu et al., 2019). Regularly arranged objects are overestimated and clustering of objects leads to underestimation (Ginsburg, 1991; Ginsburg & Goldstein, 1987). This effect of spatial organisation is particularly strong in the visual periphery where spatial pooling is more active (Valsecchi et al., 2013). Additionally, spatial grouping between objects, but not grouping by similarity between them, leads to underestimation (Yu et al., 2019). Third, spatial factors unrelated to numerosity, such as object size, total surface area, and occupied area, affect estimation (Gebuis et al., 2016; Ginsburg & Nicholls, 1988; Hurewitz et al., 2006). Finally, as with subitizing, estimation is impaired when stimuli are concentric objects (Saltzman & Garner, 1948). Thus, spatial processes play a significant role in estimation.

Nevertheless, little is known about how enumeration, whether subitizing or estimation, differs across spatial locations in the visual field. Here, we investigated if enumeration exhibits any visual field anisotropies. The default assumption is that enumeration would be uniform across the visual field, at least for locations of comparable eccentricities. However, most visual tasks, such as those measuring detection, contrast sensitivity, orientation discrimination, line-bisection, visual acuity, and efficient and inefficient visual search exhibit visual field asymmetries. Specifically, performance in these tasks is better along the horizontal meridian than along the vertical meridian, known as the Horizontal-Vertical Asymmetry (HVA; Barbot et al., 2021; Carrasco et al., 2001; Mackeben, 1999; Westheimer, 2005). Similarly, performance is better along the lower vertical meridian than along the upper vertical meridian, known as Vertical Meridian Asymmetry (VMA; Barbot et al., 2021; Carrasco et al., 2001). It has been argued that these asymmetries originate in early, low-level visual constraints, which can include meridional differences in optical projections (Zheleznyak et al., 2016), cone densities (Curcio et al., 1990; Song et al., 2011), retinal ganglion cell densities (Banks et al., 1991; Kupers et al., 2022), LGN and V1 neuron densities (Benson et al., 2021; Connolly & Essen, 1984; Kupers et al., 2022; Van Essen et al., 1984) or the number of striate and extrastriate neurons dedicated to processing different regions of space (Benson et al., 2021; Himmelberg et al., 2022; Silva et al., 2018). For example, the strength of behavioural asymmetries correlates well with asymmetries in the size of area V1 dedicated to different regions of the visual field (Himmelberg et al., 2022). While recent modelling studies have argued that cortical contributions to visual field asymmetries outweigh those by pre-cortical constraints, such as retinal cone distributions or asymmetries in ganglion cell densities (Benson et al., 2021; Kupers et al., 2019, 2022), it is possible that they arise early in the visual system and are amplified by cortical processes. Overall, these anisotropies appear to be inherent in the architecture of the visual system. Hence, one possibility is that enumeration inherits these early, low-level asymmetries.

On the other hand, the asymmetries in enumeration could be driven by downstream mid-level mechanisms underlying it. For example, while initial proposals argued that subitizing was a pre-attentive, early process (Trick & Pylyshyn, 1994), recent evidence suggests that a mid-level mechanism, attention, is crucial for individuation (Burr et al., 2010; Chakravarthi & Herbert, 2019; Ester et al., 2012; Mazza et al., 2013; Mazza & Caramazza, 2015; Olivers & Watson, 2008; Vetter et al., 2008; Xu & Chun, 2009). If that is the case, then subitizing (and perhaps other enumeration processes) should demonstrate visual field asymmetries observed in attentional tasks.

The resolution of attention is better in the lower visual field than in the upper visual field (Intriligator & Cavanagh, 2001). Performance in attentional tasks (e.g., cued letter recognition) is also better along the horizontal meridian than along the vertical meridian (Mackeben, 1999). Thus, attention too can be considered to display both HVA and VMA. Some have, however, argued that these asymmetries in attentional tasks are not characteristic of attention itself, but are instead inherited from low-level constraints (Carrasco et al., 2001). For example, manipulation of exogenous or endogenous attention does not eliminate or modify the asymmetries, suggesting that they do not originate with attention (Carrasco et al., 2001; Purokayastha et al., 2021). Nevertheless, in addition to the above, while selective attention appears to work equally well in both left and right visual fields (Arguin et al., 1990; Corbetta et al., 1993; Yamaguchi et al., 1994), there is evidence that attentional selection and processing of stimuli is better along the left horizontal meridian, and the left visual field in general, than along the right horizontal meridian or the right visual field (Asanowicz et al., 2013; Evert et al., 2003; Goodbourn & Holcombe, 2015; Hogendoorn et al., 2010; Matthews & Welch, 2015; Newman et al., 2017; Verleger et al., 2011). This asymmetry is called the Horizontal-Meridian Asymmetry (HMA; and Left Visual Field Advantage, where appropriate). For example, attention can process an object on the left horizontal meridian more accurately and with higher precision than one on the right (Hogendoorn et al., 2010). Further, performance in attentional tasks is known to exhibit a bilateral advantage, where attending to two locations across the vertical midline is easier than attending to two locations on the same side of the midline (Alvarez & Cavanagh, 2005; Chakravarthi & VanRullen, 2011; Reardon et al., 2009). While not all attentional tasks demonstrate a bilateral advantage, particularly when no distractors are present (Reardon et al., 2009), such an advantage in spatial tasks often indicates an attentional origin (Strong & Alvarez, 2020). Thus, to the extent that enumeration processes rely on attention, one might expect them (particularly, subitizing) to demonstrate these asymmetries.

We also know that other mid-level tasks present their own specific set of asymmetries. For example, visual crowding, a phenomenon where an object’s identification is impaired by nearby flankers (Bouma, 1970; Stuart & Burian, 1962), displays a characteristic set of visual field asymmetries. Since crowding affects subitizing (Chakravarthi & Herbert, 2019; Intriligator & Cavanagh, 2001) and leads to underestimation of larger numerosities (Valsecchi et al., 2013), we might expect subitizing and estimation to demonstrate the set of asymmetries peculiar to crowding. Crowding is worse along the upper vertical meridian than along the lower (VMA; Greenwood et al., 2017; He et al., 1996; Intriligator & Cavanagh, 2001; Kurzawski et al., 2021). Similarly, crowding is stronger and the range of interference between objects is larger along the vertical meridian than on the horizontal meridian (HVA; Greenwood et al., 2017; Kurzawski et al., 2021; Toet & Levi, 1992). Thus, crowding displays the same asymmetries as many low-level visual tasks and attention. Further, like attention, crowding also demonstrates a bilateral advantage (Chakravarthi & Cavanagh, 2009), where it is easier to identify two crowded targets separated by the vertical meridian compared to when they are separated by the horizontal meridian. On the other hand, crowding is worse on the left horizontal meridian than on the right (HMA; Kurzawski et al., 2021), which is the opposite of that observed in attentional tasks. To the extent that crowding influences enumeration processes, we might expect the latter to also demonstrate the asymmetries observed in crowding.

In summary, both low-level tasks and mid-level processes typically demonstrate the HVA and VMA. However, midlevel processes also exhibit an additional asymmetry, the HMA. There is no evidence, or at best poor evidence, for an HMA in low-level visual tasks such as detection, discrimination and acuity (Barbot et al., 2021; Westheimer, 2005; Yeshurun & Carrasco, 1999). Interestingly, crowding and attention exhibit opposite kinds of HMA. Performance under crowded conditions is better on the right horizontal meridian (Kurzawski et al., 2021), whereas attentional selection seems to be better on the left horizontal meridian (e.g., Goodbourn & Holcombe, 2015; Hogendoorn et al., 2010). Thus, unlike HVA and VMA, the HMA might be an idiosyncratic signature of mid-level visual processes.

Currently, it remains unclear whether any asymmetries exist in enumeration. The only documented effect of spatial location on enumeration is the bilateral advantage; while an initial study found a bilateral advantage only for estimation but not subitizing (Delvenne et al., 2011), later studies demonstrated a bilateral advantage for both subitizing (Pryor & Howe, 2015; Railo, 2014) and estimation (Railo, 2014). Another asymmetry was tentatively identified by a study that examined only subitizing (Lakha & Humphreys, 2005). In this study, a VMA was observed with better performance when stimuli were presented below fixation. However, this VMA was noticeable only for moving objects in the presence of static distractors, but not for static or moving objects in the absence of distractors. Thus, this asymmetry might not be a general feature of enumeration processes. Further, this study used a dual-task paradigm (an additional letter recognition task at fixation) and it is unclear if the findings would apply to a purely enumeration task or if they were driven by severely limited attentional resources available to the enumeration task.

In this study, we conducted two experiments to examine visual field asymmetries in enumeration. In the first, we sought to ascertain if enumeration differed by visual field locations at all or if it was uniform across (isoeccentric) locations. To do so, we tested enumeration at four cardinal locations (up, down, left, right) as well as inter-cardinal (diagonal) locations of the visual field. In the second, we systematically examined each of the asymmetries discussed above for both subitizing and estimation processes. Several distinct outcomes with associated interpretations are possible: Enumeration might not display any anisotropy (the default, although unlikely, possibility) or it might display asymmetries. These asymmetries might be inherited from low-level constraints (HVA and VMA, without an HMA) or be driven by mid-level processes such that they appear (a) attention-like (HVA, VMA and HMA where performance on the left horizontal meridian is superior to the right), or (b) crowding-like (HVA, VMA and HMA where performance on the right horizontal meridian is superior to the left). Finding patterns that match asymmetries in crowding or attention does not imply that enumeration processes rely on them, although parsimony might direct our theorising towards those processes. Such a pattern will nevertheless suggest that mid-level processes play a role in shaping enumeration processes.

## Experiment 1

### Method

#### Participants

Twenty participants (seven females) with a mean age of 21.9 years (SD = 1.7) took part in the experiment. All had normal or corrected-to-normal vision and provided written informed consent. Ethical approval was obtained from the Psychology Ethics Committee, School of Psychology, University of Aberdeen.

#### **Material and stimul**i

Participants were seated 50 cm from a 19-in. CRT screen (Sony Trinitron GDM-F520, Sony Corporation, Tokyo, Japan) with a frame rate of 100 Hz, resolution of 1,024 x 768 pixels, and 22.5 pixels per degree of visual angle. Participants’ head position was secured with a chin rest. The experiment was run using MATLAB with Psychtoolbox extensions (Brainard, 1997; Kleiner et al., 2007; Pelli, 1997).

**Fig. 1:**
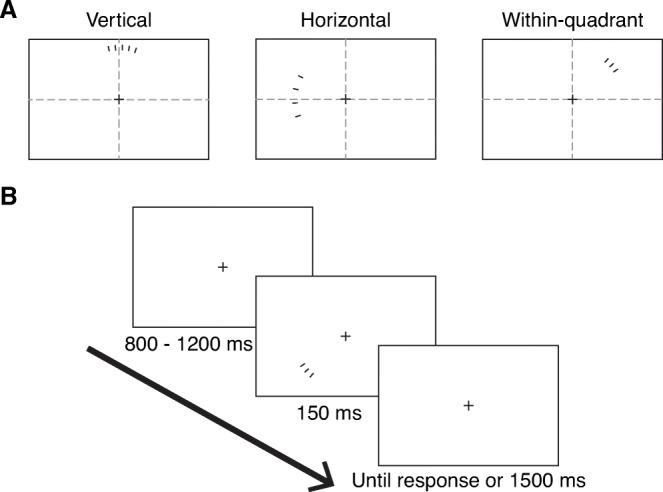
**A** Examples of stimuli displayed along the vertical meridian, horizontal meridian, or within a visual quadrant. The dashed lines depicting the vertical and horizontal meridians are for illustration purposes and were not visible during the experiment. Note that the stimuli presented across the horizontal meridian are shown for the far spacing condition, whereas the line segments are closely spaced in the other two examples. **B** Trial protocol: After a fixation period of 800–1,200 ms, 1–6 lines were presented for 150 ms in one of eight locations. Participants had up to 1,500 ms from stimulus onset to report the number of lines via a number pad

A black fixation cross of size 0.3^o^ on a white background was present throughout a block. Stimuli consisted of 1–6 black lines of height 1^o^ and width 0.25^o^ displayed on an imaginary circle of radius 7^o^ around the fixation cross (Fig. 1). All lines were oriented perpendicular to the circumference of this circle. The imaginary circle was divided into 48 equidistant points with the requirement that none of the points fall on the horizontal or vertical meridians. The points closest to the meridians were 0.45^o^ on either side of the meridian. The line stimuli were presented only at these 48 points. To test the effect of crowding, the spacing between the lines was manipulated by presenting them either on adjacent points (centre-to-centre distance of 0.9^o^) or two points apart (centre-to-centre distance of 1.8^o^). Either spacing is much higher than the two-dot resolution at that eccentricity (Anstis, 1974; Foster et al., 1989). Thus, enumeration performance would not be limited by acuity. At the closer spacing the lines would fall within crowding distance of each other, but at the larger spacing, they would be at or near the edge of the crowding region (Bouma, 1970; Toet & Levi, 1992). Note that crowding regions vary by locations around the visual field and on an individual basis (Chakravarthi et al., 2021; Greenwood et al., 2017; Kurzawski et al., 2021; Petrov & Meleshkevich, 2011); hence a fixed distance such as that used here would likely lead to some amount of crowding in some participants and locations, but not others. With that caveat in mind, we expect that stimuli at the far spacing should experience minimal to no crowding. The line stimuli were presented in one of eight peripheral locations (Fig. 1A): they straddled the upper vertical meridian, lower vertical meridian, left horizontal meridian, right horizontal meridian, or were contained completely within a quadrant (upper-left, upper-right, lower-left, or lower-right). When the number of lines presented along a meridian were even, they were distributed equally on both sides of the meridian. If the number was odd, one side of the meridian had one more line than the other, and the side with the higher number was determined randomly. When the lines were presented within a quadrant, one of the two points closest to the centre of the arc in that quadrant was randomly chosen, and the first line was placed there. The remaining lines were placed on either side of this line.

#### Procedure

Participants first took part in two practice tasks. In the first task, they were shown a black fixation cross followed by a digit between 1 and 6. Participants were asked to report the number by pressing the respective key on a numpad in order to familiarise themselves with the numpad. Each digit was presented ten times. Order of presentation of the digits was randomised. In the second practice task they were asked to complete a single block of 36 trials, which was identical to the blocks in the main experiment. On each trial (Fig. 1B) a blank screen with the fixation cross was presented for a random duration between 800 and 1,200 ms. One to six black lines were then presented at one of the eight visual field locations for 150 ms. Note that this presentation time is too brief to make eye movements, particularly since the location of targets is randomised. Eye position was therefore not monitored with an eye-tracker, but instead participants were instructed to fixate the central cross, as this would minimise errors. Participants were asked to report the number of lines within 1,500 ms from the onset of the stimulus. Failure to respond within that time window was considered an incorrect response. No feedback was provided. The next trial started right after the participant’s response or 1,500 ms after stimulus onset, whichever was earlier.

The experiment consisted of 20 blocks of 36 trials each. For each numerosity (1–6) and spacing (close, far), each of the four locations on the meridians was tested on ten trials and each of the four locations within a quadrant was tested on five trials, thus totalling 720 trials. To explore visual field asymmetries, we pooled data across specific locations. Trials across the two vertical locations (upper and lower vertical meridians) were pooled into the ‘vertical’ condition; similarly, trials from the two horizontal locations (left and right) were pooled into the ‘horizontal’ condition; finally, trials from the four quadrants (upper-left, upper-right, lower-left, or lower-right) were pooled into the ‘within-quadrant’ condition. Each of these three location conditions (vertical, horizontal, within-quadrant), therefore, had 20 trials per numerosity and spacing combination.

#### Data analysis

**Fig. 2:**
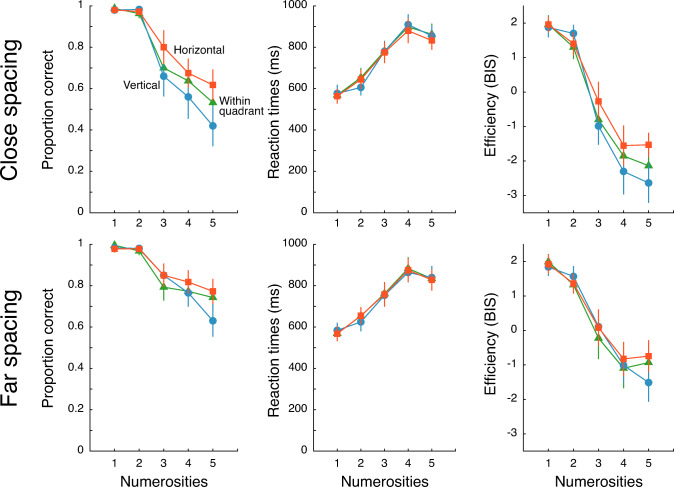
Performance in reporting numerosities in Experiment 1. In each graph, enumeration performance is plotted as a function of numerosity at each of the three location conditions (blue circles: vertical meridian; green triangles: within-quadrant; red squares: horizontal meridian) at each spacing (top row: close spacing; bottom row: far spacing). Each plot shows the means of a different measure of performance (left: accuracy; middle: median correct reaction times; right: efficiency measure BIS). Positive values for the BIS indicate better than average performance and negative values indicate worse than average performance. Error bars represent 95% confidence intervals

Trials with the highest numerosity (6) were excluded from analysis to avoid the ‘end effect’, where performance for the highest tested numerosity is often better than expected due to participants’ bias to report that number under uncertainty (Piazza et al., 2002). The initial intention for data analysis was to extract the subitizing capacity (the number of objects that can be accurately and rapidly individuated) at each location and spacing (crowding) conditions. To this end, we had planned to fit bilinear or exponential functions to accuracy data (Chakravarthi & Herbert, 2019; Ester et al., 2012). However, this could not be done with our data. Accuracy was at or near ceiling for numerosities 1 and 2, as expected, and substantially worse for 3 and higher numerosities, indicating that the subitizing capacity was at least 2 in all conditions (Fig. 2a). Crucially, the reduction in accuracy for numerosities 3–5 was shallow in some conditions but not in others. Fitting bilinear or exponential functions would lead to an underestimation of capacity in conditions with shallow slopes; such fits would produce subitizing capacities of less than 2 even when performance was near perfect at numerosity 2. For example, the subitizing capacity for the horizontal location condition determined by bilinear fitting was 1.6, whereas performance was near ceiling at numerosity 2. Hence, we decided to average performance across numerosities to obtain a bird’s eye view of differences in enumeration across visual field locations and spacing conditions. We tested a larger range of numerosities in Experiment 2 that allowed us to extract the subitizing capacity with bilinear fits.

We recorded both enumeration accuracy and reaction times on each trial. Since these two measures are known to trade off against each other and/or reflect different limitations (e.g., Heitz, 2014), we computed a single ‘efficiency’ measure that combines both behavioural observations. There are several measures of efficiency, with the Inverse Efficiency Score (IES) being the oldest and most used metric (Townsend & Ashby, 1983). However, recent studies have indicated that the Balanced Integration Score (BIS) is more robust to speed-accuracy trade-offs and changes in strategy (Liesefeld & Janczyk, 2019). Importantly, it places equal weight on both reaction times and accuracies, and is not affected by accuracy levels in any given condition, unlike previous measures of efficiency. Conceptually, it is quite simple: it is the difference between standardised accuracies and mean correct reaction times:

1 $$ {BIS}_{i,j}={z}_{i,j}(accuracy)-{z}_{i,j}(RT) $$

for the *i*^th^ participant’s *j*^th^ condition, where the z-scores are not computed as per the usual definition but as:

2 $$ {z}_{i,j}(m)=\frac{m_{i,j}-\overline{m}}{s_m} $$

That is, the z-score of measure *m* (in condition *j* of participant *i*) is the difference between participant *i*’s performance on that measure in condition *j* and the mean of that measure across all participants and conditions, standardised by the standard deviation of the observations across all participants and conditions. BIS of zero means that the efficiency in that condition was ‘average’ relative to the other conditions in the study. A positive value indicates better than average performance and a negative value indicates worse than average performance. The higher the efficiency score, the better the performance in that condition. We also computed and analysed the IES, which revealed the same pattern of results (see Online Supplementary Materials (OSM)).

To assess differences in performance across locations, we averaged performance (accuracies, RTs, or efficiency) across the tested numerosities 1–5 for each of the three location conditions separately. We then subjected these averages to a two-way repeated-measures ANOVA with visual field location (vertical, horizontal, within-quadrant) and inter-element spacing (close, far) as factors.

### Results

As expected, accuracy in reporting the number of lines decreased and corresponding reaction times increased as a function of numerosity (Fig. 2). This was also reflected in the efficiency measure that combined both accuracies and reaction times. Figure 2 (left column) shows that accuracy was high for numerosities 1–2 but was substantially worse for numerosities 3–5 in each location condition at both tested spacings. This pattern of accuracy results indicates that the subitizing capacity, the number of efficiently enumerated objects, is likely to be above 2 and less than 3 across all locations and spacings. This capacity is comparable to that found by Chakravarthi and Herbert (2019), where objects closer to each other than the maximal crowding distance (Pelli & Tillman, 2008; Toet & Levi, 1992) had a subitizing capacity of about 2. Under non-crowded conditions in the periphery or with foveal presentation, subitizing capacity tends to be higher, usually between 3 and 5 (Chakravarthi & Herbert, 2019; Kaufman et al., 1949; Trick & Pylyshyn, 1994; Warren, 1897). The low capacities documented here are likely due to mutual interference among relatively closely spaced objects.

A peculiar observation from the plots is that reaction times to numerosity 2 seem to be faster at vertical locations than at other locations. This may reflect a bilateral advantage when each line is on either side of the vertical meridian. This advantage might arise from either independent attentional resources for each hemisphere or from reduced crowding from across the vertical meridian (this latter benefit would not accrue if there are more than two lines). However, this result needs to be replicated before further speculation is in order.

#### Accuracy

**Fig. 3:**
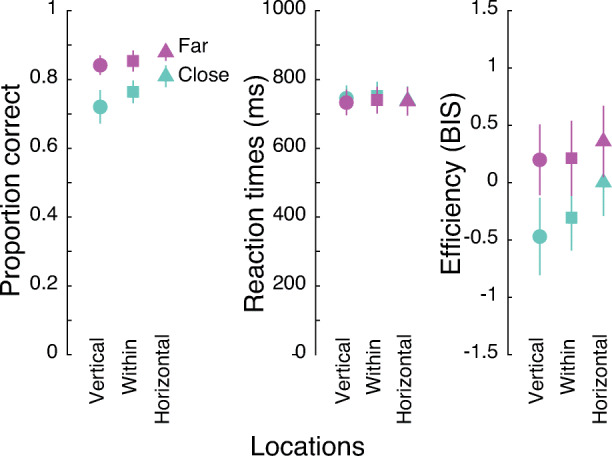
Mean enumeration performance in Experiment 1. Each panel plots the means of a different measure: accuracy (left panel), median correct reaction times (middle), and efficiency (right) at the three location conditions and two spacings. Performance for close objects is depicted in turquoise and that for far objects is depicted in purple. Error bars represent 95% confidence intervals

Enumeration accuracy (Fig. 3; see OSM Table 1 for numbers) was affected by the location of the stimuli (F(2, 38) = 13.75, p < .001, _p_η^2^ = 0.42). Post hoc tests (FDR corrected; Benjamini & Hochberg, 1995) indicated that accuracy was worst along the vertical meridian, intermediate at within-quadrant locations, and best at locations along the horizontal meridian (within-quadrant vs vertical: t(19) = 2.75, p = .025, corrected-p = 0.025, Cohen’s *d* = 0.62; horizontal vs. vertical: t(19) = 4.49, p < .001, corrected-p < .001, Cohen’s *d* = 1; horizontal vs. within: t(19) = 3.02, p = .007, corrected-p = .011, Cohen’s *d* = .67). Accuracy was higher for objects separated by a larger distance than for objects close to each other (F(1,19) = 55, p < .001, _p_η^2^ = 0.74), as observed previously in studies of enumeration and crowding (Chakravarthi & Herbert, 2019). These main effects of visual field location and spacing were qualified by an interaction (F(2,38) = 4.19, p = .023, _p_η^2^ = 0.18). This interaction reflects the finding that the differences among locations was stronger when objects were close to each other than when they were farther apart. At close spacing, enumeration at all locations differed from each other (one-way repeated-measures ANOVA: F(2,38) = 12.59, p < .001, _p_η^2^ = 0.4; pairwise comparisons: vertical vs, horizontal: t(19) = 3.99, p < .001, corrected-p = .0023, Cohen’s *d* = .89; within vs. horizontal: t(19) = 2.81, p = .011, corrected-p = .011, Cohen’s *d* = .63; vertical vs. within: t(19) = 3.2, p = .005, corrected-p = .0075, Cohen’s *d* = .71). On the other hand, when objects were farther apart, only objects located along the vertical meridian were less accurately enumerated than objects along the horizontal meridian (one-way repeated-measures ANOVA: F(2,38) = 5.35, p = .009, _p_η^2^ = 0.22; pairwise comparisons: vertical vs. horizontal: t(19) = 3.33, p = .004, corrected-p = .012, Cohen’s *d* = .75; within vs. horizontal: t(19) = 1.94, p = .067, corrected-p = .1, Cohen’s *d* = .43; vertical vs. within: t(19) = 1.16, p = .262, corrected-p = .262, Cohen’s *d* = .26).

#### Reaction times

Reaction times (RTs; Fig. 3; OSM Table 1) were roughly comparable across all the tested locations (F(2,38) = 2.8, p = .073, _p_η^2^ = 0.13), although there appears to be a trend towards differences across locations, driven by slightly slower RTs for enumeration at within-quadrant locations (none of the pairwise comparisons survive corrections for multiple comparisons; all ps > 0.08). Further, RTs were slightly faster at far spacing compared to close spacing (F(1,19) = 7.69, p = .012, _p_η^2^ = 0.29). There was no interaction between visual field locations and inter-object spacing (F(2,38) = 0.84, p = .441, _p_η^2^ = 0.04).

RTs should be considered with caution as the number of trials that are used for computing the median RT decreases with numerosity and can be as few as 6–10 trials for the largest numerosity at some locations. A lack of difference across locations according to this measure might be genuine or might reflect large variance due to few trials.

#### Efficiency (BIS)

Efficiency measures followed the same pattern as accuracy (Fig. 3; OSM Table 1). Efficiency of enumeration was modulated by visual field locations of the stimuli (F(2,38) = 12.34, p = < .001, _p_η^2^ = 0.39). Efficiency was higher at horizontal locations than at vertical (t(19) = 4.14, p < .001, corrected-p = .0017, Cohen’s *d* = 0.93) or within-quadrant (t(19) = 3.36, p = .003, corrected-p = .0045, Cohen’s *d* = 0.75) locations. However, the difference between efficiencies at the vertical and within-quadrant locations did not reach significance (t(19) = 1.77, p = .093, corrected-p = .093, Cohen’s *d* = .4). Efficiency was better for far spaced objects than for closely spaced objects (F(1,19) = 63.47, p < .001, _p_η^2^ = 0.77). These main effects were qualified by an interaction (F(2,38) = 4.45, p = .018, _p_η^2^ = 0.19). This interaction indicated that, once again, the effect of spacing was greatest along the vertical meridian than along the horizontal, and intermediate for the within-quadrant locations.

#### Summary

We place higher credence on accuracy data among the three measures. These data show that enumeration was best along the horizontal meridian, followed by within-quadrant locations and worst along the vertical meridian. This pattern was also visible with the efficiency measure, which was primarily driven by accuracy data since RTs did not seem to differ across locations. Interestingly, the difference in performance among the three location conditions was more evident for closely spaced stimuli than for those that were farther from each other. That is, crowding did not just worsen overall enumeration performance but either led to or enhanced the differences in enumeration across locations. Together, these results demonstrate visual field asymmetries for enumeration.

In a second experiment we examined if and how distinct enumeration processes, specifically subitizing and estimation, are modulated by visual field locations. We tested the three asymmetries mentioned in the introduction (HVA, VMA and HMA) with a larger range of numerosities and a larger number of trials at each of four cardinal locations.

## Experiment 2

### Method

#### Participants

Sixteen participants (12 females) with a mean age of 27.9 years (SD = 5.9), took part in the experiment. The first and second authors participated in this experiment. The rest were naïve to the purpose of the experiment. All had normal or corrected-to-normal vision and provided informed consent. Ethical approval was obtained from the Psychology Ethics Committee, School of Psychology, University of Aberdeen.

#### Material and stimuli

**Fig. 4:**
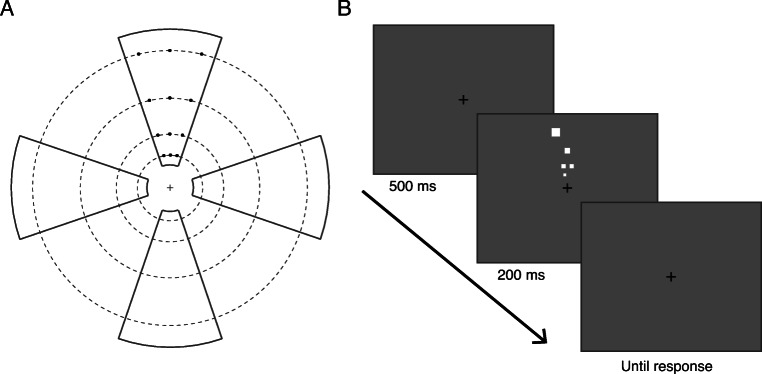
Stimulus setup and protocol of Experiment 2. **a** Illustration of possible target locations. On each trial, targets were presented within one of the four regions indicated by the black wedges along the meridians. Within these wedges, targets were presented at one or more of 12 possible locations, indicated here by black dots within the upper meridian wedge. A small eccentricity-dependent jitter was added to each location. Similar locations were also specified within the other wedges (see the section on stimuli for more details). During the experiment, the wedges, the dashed circles, and the black circles were not visible. **b** Sequence of events in a trial. A blank screen with a fixation cross was presented for 500 ms, after which a specified number of squares were presented in one of four locations (upper, lower, right or left) for 200 ms. Participants were asked to press a space bar as soon as they knew how many squares were presented and subsequently report them through the number pad. The images are not drawn to scale

The materials were the same as in Experiment 1 except that participants were seated 57 cm from the monitor, and hence there were 28.4 pixels per degree of visual angle. A black fixation cross of size 0.25^o^ on a neutral grey background was present throughout a block. Stimuli consisted of 1–9 white squares. Enumeration of these square stimuli was tested at four locations: upper, lower, left and right visual fields along the vertical and horizontal meridians. At each location, the squares were presented within a wedge-shaped region whose narrow part was close to the fovea and its wider part was towards the periphery (Fig. 4). Within each wedge, the squares could be placed at 12 possible positions. These consisted of three isoeccentric positions at each of four eccentricities: 1.1°, 1.8°, 3° and 5°. That is, the three positions were on an arc of constant radius from the fixation, with the middle position on the meridian. Two adjacent positions on the arc subtended an angle of 15° at the fixation. The linear distance between two adjacent positions at each eccentricity was 0.29°, 0.47°, 0.79° and 1.31°, respectively. These eccentricities and spacings were chosen to minimise or eliminate crowding between any two adjacent stimuli. Each position is around or on the edge of the crowding limit (half the eccentricity along the radial and quarter the eccentricity along the tangential direction) of the immediately adjacent position (Toet & Levi, 1992). Note that the more foveal (‘inner’) flanker along the radial direction is weaker than the more peripheral (‘outer’) flanker and can be placed within the half-eccentricity limit without leading to crowding (Chakravarthi et al., 2021; Petrov et al., 2007). On each trial, one of the four wedge locations was chosen. Within that wedge, a specified number of positions (the tested numerosity on that trial) was randomly selected from the 12 available positions. A small horizontal and vertical jitter of ±0.05°, 0.07°, 0.1°, 0.15° was randomly added to these positions, respectively across the eccentricities. These positions were then filled with squares whose sides were scaled across eccentricities to account for cortical magnification: 0.14°, 0.2°, 0.3°, 0.45°, respectively.

#### Procedure

Participants first took part in a practice task where they were asked to complete a single block of 72 trials, which was identical to the blocks in the main experiment. On each trial (Fig. 4b) a blank screen with the fixation cross was presented for 500 ms. One to nine white squares were then presented at one of the four visual field locations (i.e., along the upper, lower, left, or right meridian) for 200 ms. Participants were asked to press the space bar as soon as they knew, to the best of their ability, the number of presented squares and then subsequently report that number through the number pad. No feedback was provided. The next trial started 300 ms after the numerical response. Participants completed two 1-hour sessions consisting of 15 blocks of 72 trials each (1,080 trials per session). Each numerosity (1-9) at each of the four locations on the meridians was tested with 60 trials, thus totalling 2,160 trials per participant.

#### Data analysis

**Fig. 5:**
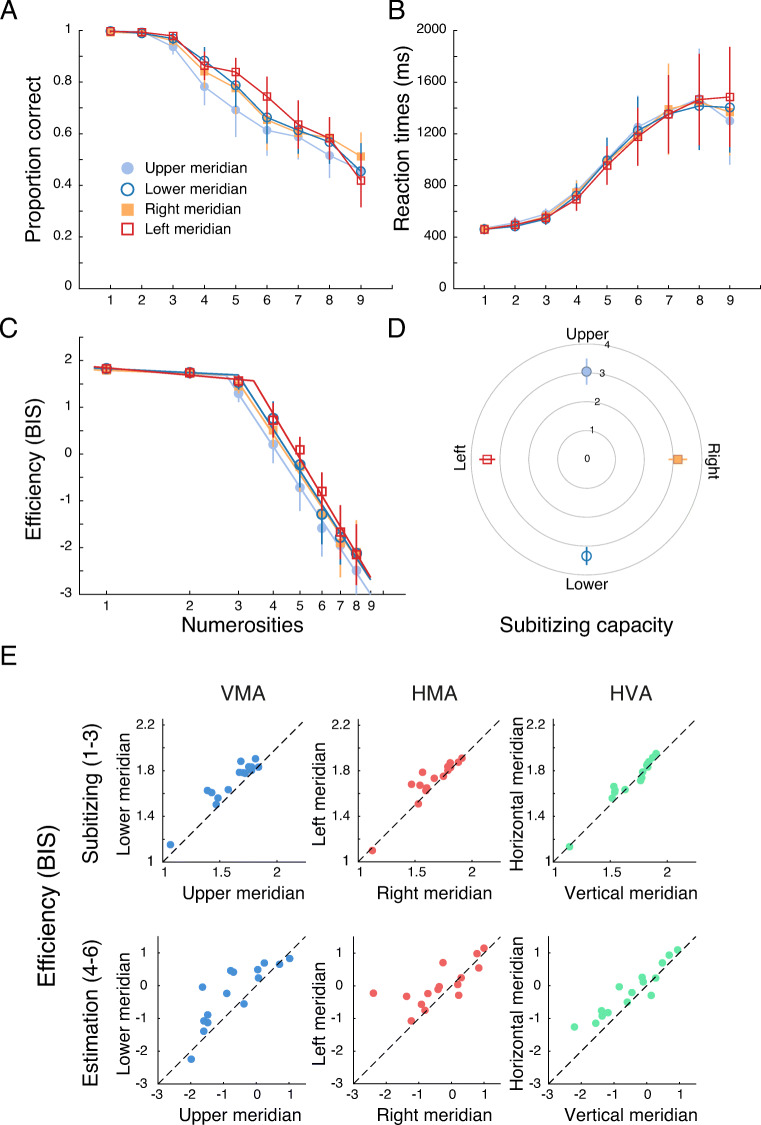
Results of Experiment 2. **a** Accuracy for enumerating briefly presented squares are plotted as a function of numerosity at the four tested locations: upper (filled pale blue circles), lower (open dark blue circles), right (filled orange square), and left (open red square) meridians. **b** Same as A for the mean of median correct reaction times as a function of numerosity. **c** Efficiency, computed as the Balanced Integration Score (BIS), plotted as a function of numerosity (log scale) separately for each location. Higher values of BIS represent higher efficiency. Bilinear fits to these data are also shown. **d** Subitizing capacity, the numerosity at which the two lines in the bilinear fits in C intersect, is depicted for each location. Error bars represent 95% confidence intervals in all plots. **e** Scatterplots of efficiency averaged over subsets of numerosities to illustrate the three asymmetries. The top row represents efficiency of subitizing (1–3 objects) and the bottom row plots efficiency of estimation (4–6 objects). Note the difference in scale for the two ranges. Individual participants’ data are plotted as circles. Each dashed line is the equality line, indicating no asymmetry. If participants’ data fall on one side of this line, it indicates the presence of an asymmetry. *VMA* Vertical Meridian Asymmetry, *HMA* Horizontal Meridian Asymmetry, *HVA* Horizontal Vertical Asymmetry

As in Experiment 1, we excluded the highest numerosity from all analyses to avoid the ‘end effect’. Reaction times were computed from the onset of the stimulus to the space bar press. Then, for each participant, we first determined enumeration accuracy and median correct reaction times at each numerosity and visual field location (upper, lower, right and left) separately. We then calculated efficiency (BIS) based on the accuracies and reaction times. To extract the subitizing capacity for each location, we fit bilinear functions (Chakravarthi & Herbert, 2019; Ester et al., 2012) to these efficiency data as a function of log10(numerosity). We allowed the intercept and slopes of both line segments to vary (Fig. 5c). The ‘elbow’ point where the two lines intersect indicates the subitizing capacity. Numerosities less than this capacity are subitized, whereas those above need to be estimated. Mean R^2^ of these fits was 0.92, indicating that the bilinear fits described the data well.

For each enumeration process, subitizing and estimation, we assessed the presence of the three visual field asymmetries. We compared efficiency (averaged over numerosities 1–3 for subitizing and 4–6 for estimation; see subsection on Subitizing Capacity in Results below for more details) between upper and lower locations to test for VMA and between left and right locations to determine the presence of HMA. To examine HVA, we pooled trials across upper and lower locations to determine accuracy and reaction times for the ‘vertical’ condition; similarly, we pooled trials across left and right locations to determine accuracy and reaction times in the ‘horizontal’ condition. We then computed the efficiency scores (BIS) on these data. We conducted FDR corrected (Benjamini & Hochberg, 1995) pairwise t-tests to assess VMA, HMA and the HVA within each numerosity range. Analysis using the IES efficiency measure produced similar results and can be found in the OSM.

One participant’s data was excluded from analysis, since their accuracy was less than 40% for all numerosities greater than 3 and their reaction times were flat and fast, around 350 ms, for *all* numerosities, indicating that they did not follow task instructions.

### Results

Accuracy and reaction times, averaged over participants, in enumerating the presented objects are plotted in Fig. 5 (panels A and B, respectively). Plots of performance at the horizontal and vertical locations (pooled over upper and lower locations, and left and right locations, respectively) can be found in the OSM. Efficiency of performance, as measured by BIS, at the four visual field locations are shown in Fig. 5c. Also plotted are the bilinear fits to the efficiency data. The elbow points, representing the subitizing capacity at each location, are summarised in Fig. 5d.

#### Subitizing capacity

A one-way repeated-measures ANOVA did not reveal any differences between subitizing capacities at the four locations (F(3,42) = 2.22, p = .1, _p_η^2^ = 0.14). The capacities at all four locations are just above 3 and are comparable. Hence, we segmented the tested numerosities into two groups: 1–3 items for subitizing and 4–8 items for estimation. We then averaged efficiency scores (Table 1) for 1–3 items to assess subitizing and 4–6 items for estimation. We restricted the estimation range to 4–6 items so that the set size of the two ranges, subitizing and estimation, are the same. Using a different range for estimation, say 4–8 objects, does not change the pattern of results (see OSM).

**Table 1:** Efficiency (mean and 95% confidence interval) of subitizing and estimation at different visual field locations. Efficiency along vertical and horizontal meridians are derived from trials pooled over upper and lower locations and left and right locations, respectively

	Upper	Lower	Right	Left	Vertical	Horizontal
Subitizing (1–3)	1.61 (1.49, 1.73)	1.7 (1.59, 1.81)	1.66 (1.55, .177)	1.71 (1.6, 1.82)	1.69 (1.57, 1.8)	1.72 (1.61, 1.83)
Estimation (4–6)	-0.7 (-1.22, -0.18)	-0.25 (-0.76, 0.26)	-0.34 (-0.86, 0.17)	0.01 (-0.34, 0.35)	-0.49 (-0.99, 0.02)	-0.17 (-0.58, 0.23)

#### **Efficiency of subitizing: 1**–**3 objects**

Efficiency (Table 1, Fig. 5e) was higher for subitizing objects along the lower vertical meridian than along the upper vertical meridian (t(14) = 5.28, p < .001, corrected-p < .001, Cohen’s *d* = 1.36). Efficiency was also higher along the left horizontal meridian than along the right horizontal meridian (t(14) = 2.66, p = .019, corrected-p = .029, Cohen’s *d* = 0.69). Furthermore, efficiency along the horizontal meridian was greater than along the vertical meridian (t(14) = 2.41 p = .031, corrected-p = 0.037, Cohen’s *d* = 0.62). These asymmetries can be observed at the individual level in the scatter plots in Fig. 5e (top row).

#### Efficiency of estimation: 4-6 objects

Figure 5e (bottom row) presents scatterplots of estimation efficiency at pairs of meridional locations. Efficiency (Table 1) was once again higher for estimating objects on the lower vertical meridian than on the upper vertical meridian (t(14) = 3.18, p = .007, corrected-p = .014, Cohen’s *d* = 0.82). While efficiency of estimation was higher on the left horizontal meridian compared to the right, the difference was just above the statistical significance threshold (t(14) = 2.07, p = .057, corrected-p = .057, Cohen’s *d* = 0.53). It would be premature to claim that there is no difference in efficiency at these locations and further testing would be required to assess the extent of the difference. Finally, efficiency for estimating objects along the horizontal meridian was higher than that along the vertical meridian (t(14) = 3.62, p = .003, corrected-p = .009, Cohen’s *d* = 0.94).

In summary, we observed VMA (lower > upper) and HVA (horizontal > vertical) in both subitizing and estimation, as documented for multiple visual tasks (Barbot et al., 2021; Carrasco et al., 2001; Himmelberg et al., 2022). There was also a HMA in subitizing, and a trend towards such an asymmetry in estimation. In both cases, efficiency was higher in the left visual field than in the right.

## General discussion

The current study examined, in two experiments, visual field asymmetries in numerosity processing. The results of the first experiment demonstrated that enumeration was not uniform across the visual field; enumeration was better along the horizontal meridian than along the vertical meridian with intermediate locations exhibiting intermediate performance. It also indicated that enumeration was impaired when objects were close to each other and that this crowding effect enhanced the asymmetries. The second experiment investigated the specific pattern of visual field asymmetries. It found that subitizing capacities, the number of objects that are efficiently enumerated, were around three items and comparable across cardinal locations (up, down, left, and right). However, both subitizing and estimation demonstrated a range of asymmetries: a Vertical Meridian Asymmetry (VMA), where performance was better on the lower vertical meridian than on the upper vertical meridian; a Horizontal Vertical Asymmetry (HVA), where performance was better along the horizontal meridian than the vertical; and a Horizontal Meridian Asymmetry (HMA), where performance was better on the left horizontal meridian compared to the right.

### Role of spacing

Experiment 1 tested the effect of spacing between objects. Previous studies have shown that closely spaced objects are underestimated both in subitizing (Chakravarthi & Herbert, 2019; Sayim & Taylor, 2019) and estimation (Bertamini et al., 2016; Chakravarthi & Bertamini, 2020; Valsecchi et al., 2013). That is, inter-object spacing modulates enumeration processes. Here, we found the same effect of spacing: enumeration of closer objects was worse than that of farther objects. More importantly, the differences in enumeration across visual field locations, while observable at both tested spacings, was more prominent at the closer spacing. For example, the HVA was stronger at close spacing than at far spacing, indicating that crowding, at a minimum, magnifies existing visual field asymmetries.

The differences in enumeration performance across visual field locations appear to match the variations in crowding across the visual field. We found that enumerating objects was worst along the vertical meridian, best along the horizontal meridian, and intermediate at within-quadrant locations. Similarly, crowding is weakest along the horizontal meridian and strongest along the vertical meridian. Some studies have documented, at least in a subset of observers, intermediate strength of crowding at within-quadrant locations (Toet & Levi, 1992), although others have not (Petrov & Meleshkevich, 2011). Nevertheless, it is surprising that even when the objects were expected to experience low to minimal crowding (Chakravarthi & Herbert, 2019; Pelli & Tillman, 2008), the same pattern of asymmetries were observed (far spacing in Experiment 1; Experiment 2). This suggests that, while crowding enhances existing asymmetries, it is likely *not* at the origin of these asymmetries. Of course, it can be argued that the inter-object distances used in these experiments might still lead to some crowding, which might reproduce the crowding asymmetries. However, if crowding were the underlying cause of asymmetries, we would expect to observe an HMA where performance is better along the right meridian (Kurzawski et al., 2021), but we observed the opposite. Our results thus suggest that the visual field asymmetries that we observed in enumeration is not caused by crowding.

### Asymmetries in subitizing and estimation

Our study revealed several visual field asymmetries in enumerating objects in both subitizing and estimation regimes. Interestingly, these asymmetries were similar for both processes, with at most minor differences. These similarities suggest that the same constraints apply to both processes. Previous studies have argued that low-level visual constraints, such as differences in the allocation of neural resources in early cortical (V1) areas, are inherited by a host of visual processes, which exhibit the same types of asymmetries. Both VMA and HVA have been attributed to such low-level constraints (Barbot et al., 2021; Carrasco et al., 2001; Rovamo & Virsu, 1979), although there are claims that they might be attributable to attentional processes (Intriligator & Cavanagh, 2001; Mackeben, 1999; but see Carrasco et al., 2001; Purokayastha et al., 2021). These considerations can be taken to suggest that the asymmetries we observed are likely to have been inherited from low-level processes, without having to invoke mid-level processes such as attention or crowding.

Crucially, however, we also discovered an HMA, where enumeration performance was better on the left horizontal meridian than on the right. This asymmetry is not usually observed in visual tasks that rely on early visual processes such as acuity (Barbot et al., 2021; Yeshurun & Carrasco, 1999). Nevertheless, certain visual tasks that rely on mid-level processing exhibit the HMA. This is the case for crowding but with an HMA in the opposite direction, with worse performance on the left horizontal meridian than on the right (Kurzawski et al., 2021). On the other hand, other mid-level processes such as attention have been known to preferentially process objects on the left horizontal meridian compared to the right under some circumstances (Goodbourn & Holcombe, 2015; Hogendoorn et al., 2010). With the caveat that we likely have not surveyed the whole range of mid-level visual processes in terms of HMA, our results are consistent with the argument that the observed HMA is the consequence of visual field asymmetries driven by visual attention. That is, our results point to a role of attention in both enumeration processes, explaining the HMA.

There is considerable evidence that subitizing relies on attentional mechanisms, as discussed in the introduction (Burr et al., 2010; Chakravarthi & Herbert, 2019; Mazza & Caramazza, 2015; Olivers & Watson, 2008; Vetter et al., 2008; Xu & Chun, 2009). However, enumeration beyond the subitizing range involves the approximate number system (Dehaene, 1992; Feigenson et al., 2004). The role of attention in estimation is much more disputed. Some studies claim that it is not essential (Burr et al., 2010), while others argue that it is involved (Pomè et al., 2019, 2021). Interestingly, some studies that were not intended to test the role of attention nevertheless seem to support the contention that attention plays a role. For example, studies have shown that not only subitizing but also estimation displays a bilateral advantage (Delvenne et al., 2011; Railo, 2014), where enumeration is better when objects are split across the vertical meridian (and thus processed independently by the two cortical hemispheres) than when they are split across the horizontal meridian (and thus processed by the same cortical hemisphere). Bilateral advantage has been considered to be a marker of attention (Alvarez & Cavanagh, 2005) and hence it can be inferred that attention plays a role in estimation. Similarly, the ability to enumerate only approximately (with error increasing with numerosity) for numerosities beyond the subitizing range has been argued to be due to limited informational capacity (Cheyette & Piantadosi, 2020) and on serial accumulation mechanisms (Cheyette & Piantadosi, 2019), indicating that a central bottleneck, aka attention, underlies estimation. Thus, attention might be centrally involved in estimation processes and thus in all enumeration processes. This might account for the observed HMA in this study.

Some have argued that rapid enumeration is not subserved by two distinct processes, subitizing and estimation, but instead by a single enumeration process whose precision varies with numerosity (Cai et al., 2021; Cheyette & Piantadosi, 2020; Tsouli et al., 2022). By this argument, the observed asymmetries are simply the asymmetries of the same enumeration mechanism applied to different numerosity ranges. As noted above and in the introduction, there is an ongoing vigorous debate about whether subitizing and estimation are distinct processes (e.g., Anobile et al., 2016; Dehaene, 1992; Feigenson et al., 2004) or a single mechanism that exhibits seemingly distinct characteristics at different numerosities (Cai et al., 2021; Cheyette & Piantadosi, 2020). Our study cannot distinguish between these two accounts, but the finding that an HMA is found in both ranges is compatible with both possibilities.

An ever-present concern is whether we measure what we intend to measure. While it is clear that behavioural performance is different for subitizing and estimation, it is less clear whether numerosities above the subitizing capacity (4–9) are being *estimated*. One alternative is that participants use iconic or visual short-term memory to *count* the briefly presented objects. Given the relatively fast reaction times for higher numerosities in our study (roughly 1,200 ms), it seems implausible that participants were engaging in serial counting (Kaufman et al., 1949; Warren, 1897). However, participants could be using a mixed strategy of partial counting and estimation to improve performance. While plausible, it has been argued that under time pressure, higher numerosities (>5) are estimated (Kaufman et al., 1949; Warren, 1897). Nevertheless, even if a mixed strategy of counting and estimation were involved, the pattern of observed asymmetries can be attributed to attention, since attention is closely involved also in counting (Cavanagh & He, 2011) and can be argued to be involved in estimation (Cheyette & Piantadosi, 2019, 2020).

### Relationship to other phenomena

#### Redundancy masking

Recent experiments on enumerating objects in the periphery have highlighted a finding that identical and regularly spaced objects are underestimated, particularly when they are very close to each other (Sayim & Taylor, 2019; Yildirim et al., 2020). This has been termed ‘redundancy masking’. This underestimation disappears with increased spacing, even if the objects are close enough to crowd each other. It is also absent when objects are arranged concentrically around the fixation, as in Experiment 1 in this study, or if they are irregularly organised, as in Experiment 2. Thus, the conditions in which we tested our stimuli do not match those required for redundancy masking, suggesting that our findings are not likely to reflect asymmetries in such processes. Instead, they point to asymmetries in the enumeration process itself.

The visual field asymmetries that we observed in this study are also substantially different from those found by Yildirim and colleagues (2022) in redundancy masking. First, unlike here, they did not observe an VMA. Second, they found more underestimation (stronger ‘redundancy masking’) along the horizontal meridian than along the vertical. This pattern of worse performance along the horizontal meridian is unusual compared to most visual tasks and the opposite of the HVA found here for enumeration. The authors attributed these atypical asymmetries to differing abilities in extracting regularities or differing amounts of spatial compression across the visual field, both of which they argue are stronger along the horizontal meridian. That is, the asymmetries they observed were not thought to be due to differences in enumeration (or crowding) across the visual field, but due to a specific type of mutual interference between objects when they are organised in a particular pattern.

There are several differences in the organisation of the stimuli and protocol between our Experiment 1 and the experiments conducted by Yildirim and colleagues (2022) that might explain the discrepancy in the results, and potentially in the respective mechanisms that these paradigms evoke. First, we presented 1–6 lines and asked observers to report a number between 1–6, whereas in their study, participants were presented with 3–7 lines but were asked to report any number between 0–9. That is, participants were free to choose a number beyond the presented numerosity range, which might affect the responses provided. Second, their stimuli were much more closely spaced compared to our study (0.4–0.85° spacing between lines at a mean eccentricity of 10°; in our study inter-line spacing was about 1° and 2° at an eccentricity of 7°). This would have led to particularly strong flanker-induced interference in their stimuli compared to ours. More importantly, their lines were ‘radially’ arranged, where lines were oriented perpendicular to the straight line connecting them to fixation (and hence were at different eccentricities), whereas in our experiment, the lines were arranged ‘tangentially’ where each line was at the same eccentricity and was oriented towards the fixation. This would have led to even more interference in their stimuli than ours (Toet & Levi, 1992). Further, a previous study from the same lab had found no redundancy masking with tangential lines (Yildirim et al., 2020). These differences suggest that different processes might underlie the processing of the two sets of stimuli, even though the tasks and stimuli might appear comparable at first glance.

In summary, the asymmetries that are observed in enumeration depend on the exact underlying processes that are being assessed by the task. Redundancy masking examines a specific type of inter-object interference, and the asymmetries reflect that process. On the other hand, the asymmetries we uncovered are robust to type of stimuli (lines or squares), organisation (isoeccentric or distributed), spacing (close or far; crowded or uncrowded), response type (direct numerical response or space bar), and across the type of enumeration (subitizing or estimation). These asymmetries likely reflect differences in the enumeration process across visual field locations.

#### Oblique effect

There is, however, another potential explanation that we need to consider for the results of Experiment 1. Our stimuli had different orientations at different locations: they were always presented perpendicular to an imaginary circle centred on fixation. Objects oriented in a cardinal direction (vertical or horizontal) are processed better than those in oblique directions, the so-called ‘oblique effect’ (Appelle, 1972; Saarinen & Levi, 1995; Westheimer, 2003). It is therefore possible that enumeration is worse at some locations merely because of the orientation of the stimuli at those locations. However, this proposal cannot account for our results. As noted above, impairment of enumeration was not the highest at within-quadrant locations. In fact, enumeration performance at within-quadrant locations was better than that at vertical locations. Further, the difference in enumeration was largest between vertical and horizontal locations (and hence between vertical and horizontal orientations), even though orientation processing is not that different between horizontal and vertical orientations (Westheimer, 2005). Finally, the same HVA was observed in Experiment 2, where orientation does not play a role. These findings and arguments indicate that the pattern of results cannot be explained by the oblique effect.

## Conclusion

We observed clear asymmetries – HVA, VMA, and HMA – in both subitizing and estimation. The most parsimonious explanation for all three asymmetries is that enumeration processes are underpinned by attention and hence exhibit attention-specific asymmetries. However, our findings are also consistent with inheritance of some asymmetries (HVA, VMA) from early visual processes while other asymmetries (HMA) can be attributed to mid-level processes, such as attention. While our study cannot distinguish between these alternatives, our main take away is to emphasise the presence of specific visual field asymmetries in enumeration, which has not been documented before, and to note the effect of inter-object spacing on such asymmetries.

## Supplementary information

ESM 1 (DOCX 241 kb)
