# Supplementary material for: Visual field asymmetries in numerosity processing
Source: Atten Percept Psychophys. 2022 Oct 18;84(8):2607–22. doi: 10.3758/s13414-022-02585-1 (PMC9630184; doi:10.3758/s13414-022-02585-1)
Supplement: Supplementary file 1 — (DOCX 241 kb) [file 13414_2022_2585_MOESM1_ESM.docx]

Supplementary Materials

# Performance in Experiment 1

Supplementary Table 1: Performance in the enumeration task in Experiment 1 at the three location conditions and two spacings. Means (with 95% confidence intervals) for each of the three measures are reported.

|  | Close spacing | | | Far spacing | | |
| --- | --- | --- | --- | --- | --- | --- |
|  | Vertical | Within-quadrant | Horizontal | Vertical | Within-quadrant | Horizontal |
| Accuracy | 0.72  (0.67, 0.77) | 0.76  (0.73, 0.8) | 0.81  (0.78, 0.84) | 0.84  (0.81, 0.87) | 0.85  (0.82, 0.88) | 0.88  (0.85, 0.9) |
| RT (ms) | 745  (708, 783) | 753  (712, 793) | 739  (698, 780) | 733  (696, 769) | 740  (701, 779) | 736  (696, 777) |
| Efficiency (BIS) | -0.47  (-0.81, -0.13) | -0.31  (-0.59, -0.02) | 0  (-0.29, 0.29) | 0.2  (-0.11, 0.51) | 0.21  (-0.11, 0.54) | 0.36  (0.05, 0.67) |

# IES measure

In this study, we combined accuracy and reaction time measures to obtain a single ‘efficiency’ score to allow us to compare across numerosities and to avoid speed-accuracy trade-offs to affect the interpretation. Following the recommendation by recent studies (Liesefeld & Janczyk, 2019), we used the balanced-integration score as a measure of efficiency, which is considered a robust measure, unaffected by strategies and internal criteria. A measure that has been traditionally used, however, is the Inverse Efficiency Score (IES). Here we present the same data analysed using this measure for both experiments 1 and 2. IES for condition *j* in participant *i* is obtained by dividing the mean reaction time on correct trials by the accuracy in condition *j* and participant *i*:

$${IES}_{i,j}= \frac{\bar{{RT}_{i,j}}}{{acc}_{i,j}}$$

IES is interpreted as “the average energy consumed by the system over trials” (Townsend & Ashby, 1983, p. 204). The lower the IES number the more efficiently the participant performed in that condition.

## Experiment 1


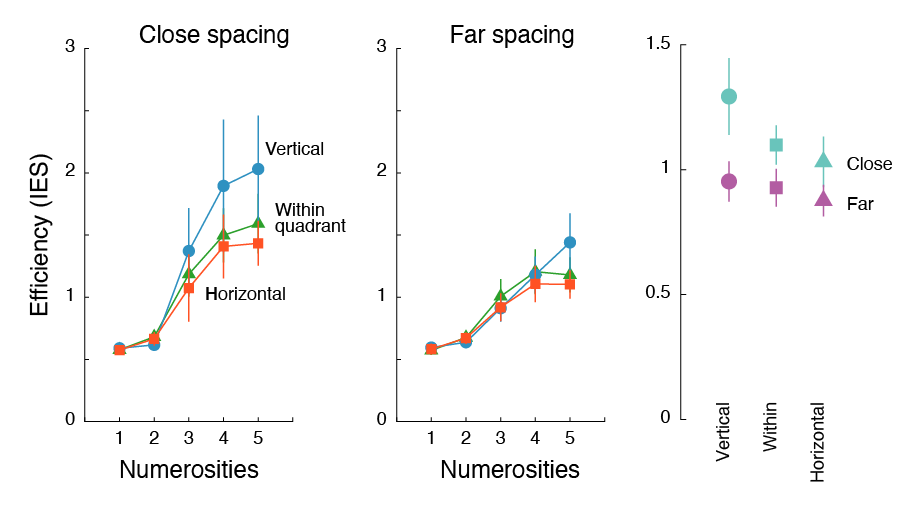


**Supplementary Figure 1**. Efficiency, IES, of performance in Experiment 1. The first two panels (left and centre) plot IES as a function of numerosity for each spacing (close and far). Each line presents data for a different location condition. Note that, unlike BIS, the lower the IES value, the more efficient the performance. The right panel plots efficiency, averaged over numerosities, for the three location conditions and two spacings. The pattern of results match those for BIS (main text). Error bars represent 95% CI.

## Experiment 2


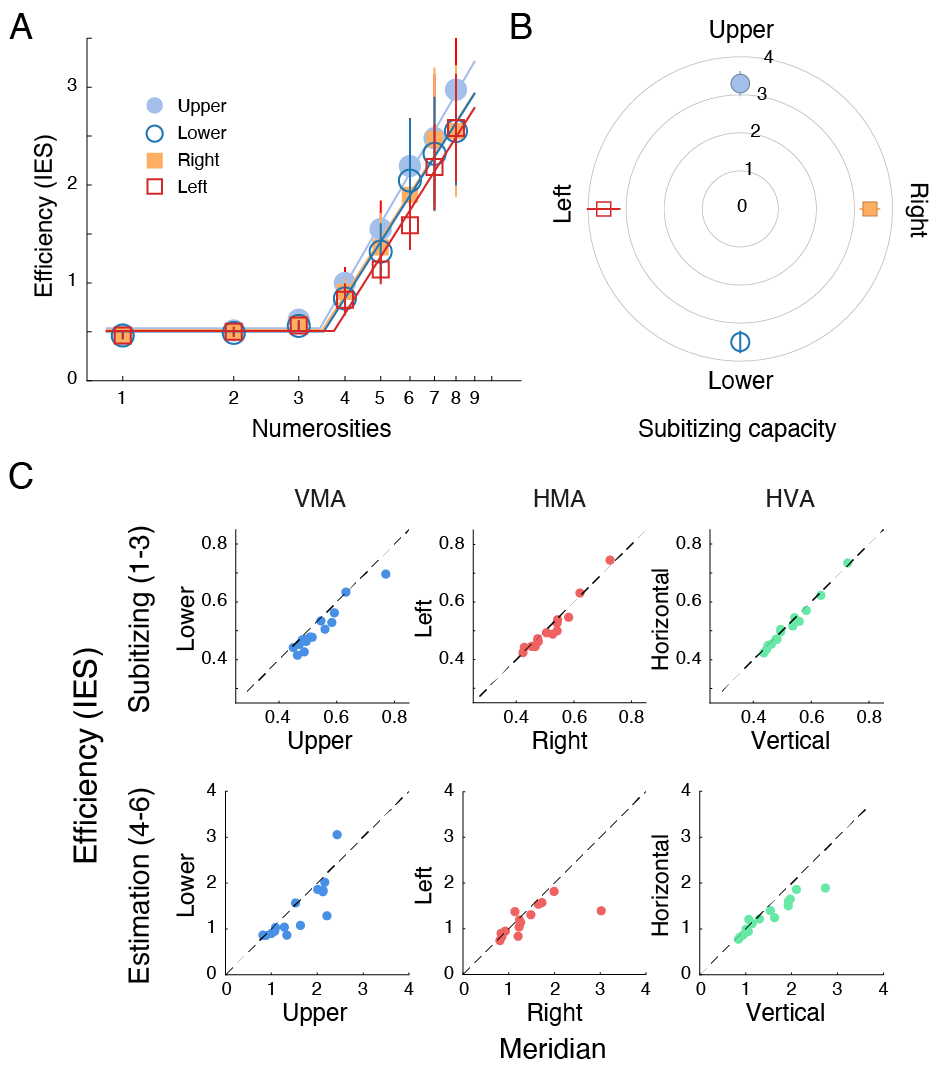


**Supplementary Figure 2**: Results of Experiment 2 analysed with the IES measure. **A**. Efficiency, computed as the Inverse Efficiency Score (IES), plotted as a function of numerosity (log scale) separately for each location. Lower values of IES represent higher efficiency. Bilinear fits to these data are also depicted. **B.** Subitizing capacity, the numerosity at which the two lines intersect in the bilinear fits in A, is depicted for each location. Error bars represent 95% CI in all plots. **C.** Scatterplots of efficiency averaged over subsets of numerosities to illustrate the three asymmetries. The top row represents efficiency of subitizing (1-3 objects) and the bottom row plots efficiency of estimation (4-6 objects). Note the difference in scale for the two ranges. Individual participants’ data are plotted as circles. Dashed line in each plot is the equality line, indicating no asymmetry. If participants’ data fall on one side of this line, it indicates the presence of an asymmetry. The results are the same as with the BIS efficiency measure. Note that the data points are on the ‘other’ side of that observed for BIS, since for IES lower values indicate higher efficiency. VMA = Vertical Meridian Asymmetry; HMA = Horizontal Meridian Asymmetry; HVA = Horizontal Vertical Asymmetry.

# Horizontal vertical asymmetry in Experiment 2

To assess the HVA, we pooled trials from the left and right locations to form a ‘horizontal’ condition and from the upper and lower locations to form a ‘vertical’ condition. Accuracy and reaction times from these pooled conditions are plotted in Supplementary Figure 3 (left and centre panels). Efficiency (BIS) computed over these pooled trials are plotted in the right panel of Supplementary Figure 3.


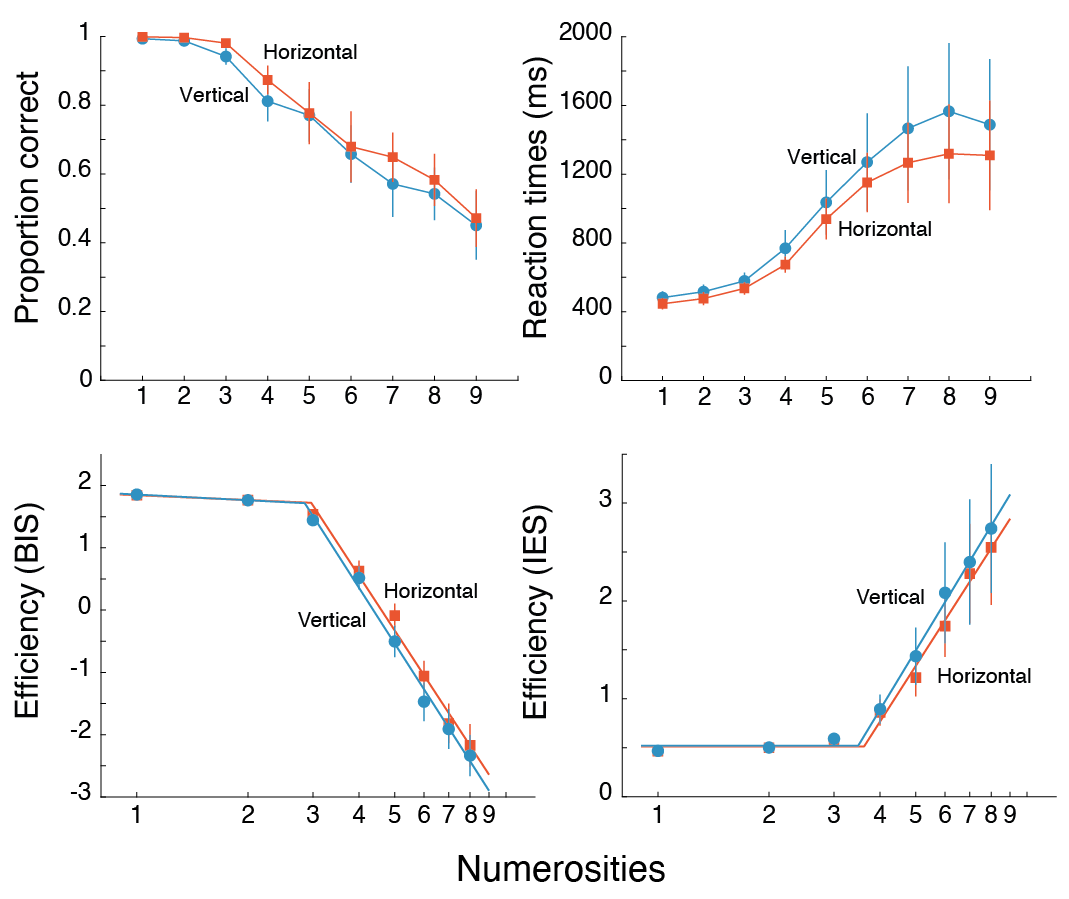


**Supplementary Figure 3**. Performance (accuracy – top left panel; reaction times – top right panel; efficiency scores, computed as BIS – bottom left panel, computed as IES – bottom right panel) plotted as a function of numerosity along horizontal and vertical meridians. Data for horizontal meridian were obtained by pooling trials over the right and left horizontal locations; data for the vertical meridian were obtained by pooling trials over the upper and lower vertical locations. Error bars are 95% CI.

# Varying the estimation range

In the main analysis, we averaged performance (efficiency) over numerosities 4-6 to assess asymmetries in estimation. This range was chosen to ensure that the set size in the estimation range was the same as in the subitizing range (averaged over 1-3 objects). Here, we varied the estimation range in different ways to determine if the specific choice of the range (4-6) uniquely led to the pattern of observed results. First, we used a larger range (4-8), which included all estimated numerosities. Note that 9 was not included because of the ‘end effect’ (Piazza et al., 2002). Second, we excluded numerosity 4, which might be considered to be on the borderline between subitizing and estimation, and it could even be within the subitizing range of some participants. Hence, we tested the range 5-7. Finally, we included numerosities that are likely to be only estimated (6-8). We find that the pattern of results remains largely unchanged from that observed for 4-6. Most data points remain off-diagonal to one side of the scatter plot, indicating consistent asymmetries, irrespective of the range used.


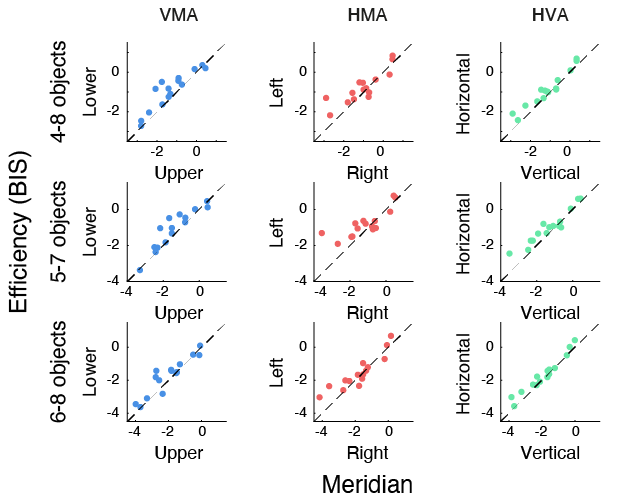


**Supplementary Figure 4**. Scatterplots of efficiency averaged over different ranges of numerosities to illustrate the three asymmetries. The top row averages over 4-8 objects; the middle row 5-7 objects and the bottom row 6-8 objects. Each dashed line is the equality line, indicating no asymmetry. If participants’ data fall on one side of this line, it indicates the presence of an asymmetry. VMA = Vertical Meridian Asymmetry; HMA = Horizontal Meridian Asymmetry; HVA = Horizontal Vertical Asymmetry.

# References

Liesefeld, H. R., & Janczyk, M. (2019). Combining speed and accuracy to control for speed-accuracy trade-offs (?). *Behavior Research Methods*, *51*(1), 40–60.

Piazza, M., Mechelli, A., Butterworth, B., & Price, C. J. (2002). Are Subitizing and Counting Implemented as Separate or Functionally Overlapping Processes? *NeuroImage*, *15*(2), 435–446. https://doi.org/10.1006/nimg.2001.0980
